# Hematological parameters as predictors of complex angiographic coronary lesions in patients with acute myocardial infarction

**DOI:** 10.1371/journal.pone.0345569

**Published:** 2026-03-27

**Authors:** Thadzia Maria de Brito Ramos, José Gildo de Moura Monteiro Júnior, Veridiana Câmara Furtado, Maria Luísa Souza de Paula, Bárbara Farias Bastos, Maria Júlia Ordônio Pires, Ulisses Ramos Montarroyos, Dário Celestino Sobral Filho

**Affiliations:** 1 Cardiological Hospital of Pernambuco – PROCAPE, University of Pernambuco – UPE, Recife, PE, Brazil; 2 University of Pernambuco (UPE), Recife, Brazil; 3 School of Medical, University of Pernambuco (UPE), Recife, Brazil; University of Diyala College of Medicine, IRAQ

## Abstract

Acute myocardial infarction is an essentially inflammatory disease. The inflammation is predictive of adverse clinical outcomes in coronary artery disease, and the Syntax Score was developed as an angiographic scoring system to anatomically classify the complexity of atherosclerosis plaques. The inflammatory response leads to an increase in mean platelet volume (MPV), neutrophil/lymphocyte ratio (NLR) and the appearance of nucleated red blood cells (NRBCs) in the peripheral blood of the patients with acute myocardial infarction. These hematological variables express systemic inflammation and hypoxia; therefore, they are markers of these tissue injuries. Severe abnormalities in these parameters are associated with a worse prognosis and were organized within a hematologic scoring system, i.e., a hematologic score. The objective of this study was to determine the sensitivity and specificity of these hematologic biomarkers in order to estimate the angiographic severity of coronary lesions. This was a secondary analysis of a larger study “Laboratory Model for Stratification of Risk of Intra-hospital Death Associating Hematological Score, Immunological Markers and Metalloproteinases in Patients with Acute Myocardial Infarction”, conducted by our research group. Individuals hospitalized with a diagnosis of acute myocardial infarction and undergoing coronary angiography during hospitalization were included in the study. Upon admission to the health service, nucleated red blood cell count, neutrophil/lymphocyte ratio, and mean platelet volume were obtained. Pregnant women, patients with oncological or hematological diseases, those with prior use of corticosteroids or chemotherapy, and those who returned to the hospital after discharge were excluded. A total of 479 patients, aged 62.3 ± 11.1 years, were studied, 63% of whom were male. In this study, the significant presence of NLR or MPV, with values ≥ 10.4 and ≥ 3.7, respectively, correlated well with a high syntax score (sensitivity 81.8%, specificity 15.9%, negative predictive value 92.2%).

## Introduction

Acute myocardial infarction is an essentially inflammatory disease [1] and inflammation predicts adverse clinical outcomes in coronary artery disease (CAD) [2]. In this context, the Syntax Score is widely used to classify the complexity and severity of atherosclerotic plaque [3,4], making it possible to evaluate several aspects related to coronary plaque, such as location in the vessel, calcification, proximity to bifurcation, chronic occlusion, among others, the anatomical complexity being classified in three ranges: 0–22 (low score), 23–32 (intermediate score), and greater than 32 (high score) [5].

Recent studies have shown that nucleated red blood cells (NRBCs), mean platelet volume (MPV) and neutrophil/lymphocyte ratio (NLR) are hematological biomarkers capable of expressing the degree of hypoxemia and systemic inflammation such as metabolic syndrome, sepsis, malignancy and CAD itself [6–8]. The evaluation of complete blood count on admission and discharge has also been shown to be useful for risk classification in individuals with acute myocardial ischemia [9], leukocyte count, red blood cell distribution width (RDW), neutrophil-lymphocyte ratio, platelet-lymphocyte ratio and a mean platelet volume (MPV) all of which are significantly higher in these patients [10].

NLR is positively associated with a higher risk of cardiovascular disease incidence and worse angiographic results [11,12]. In this scenario, the development of clinical scores related to cardiovascular diseases using components of the hemogram has been increasingly studied, for example, neutrophil, monocyte, platelet and lymphocyte counts that comprise a new index of response to systemic immunological inflammation [13]; mean corpuscular volume, mean corpuscular hemoglobin, mean corpuscular hemoglobin concentration and RDW all showed a positive correlation with the Syntax Score [14].

NRBC, NLR and MPV represent the components of the blood count. The red series (erythroblast), the white series (leukogram) and the platelet series (platelet count) of the hemogram are part of a hematological scoring system that has been shown to be an independent predictor of mortality in patients with acute myocardial infarction [15,16]. To estimate the severity of coronary lesions, we investigated the diagnostic value (sensitivity and specificity) of these hematologic biomarkers.

## Methods

### Study population

In March 20, 2023, we performed a secondary analysis of data collected from the study “Laboratory Model for Stratification of Risk of Intra-hospital Death Associating Hematological Score, Immunological Markers and Metalloproteinases in Patients with Acute Myocardial Infarction”, carried out by our research group. This secondary analysis comprised 479 patients with acute myocardial infarction admitted between June 1, 2019 and October 31, 2019 to the Pernambuco Cardiac Emergency Unit (PROCAPE), a university hospital that offers specialized diagnosis, procedures and treatments for heart disease. The following were excluded from this study: Pregnant women, patients with oncological or hematological diseases, those with prior use of corticosteroids or chemotherapy, and Individuals readmitted to the hospital after initial discharge, as well as those under the age of eighteen. The diagnosis of acute myocardial infarction was supported by the anamnesis, electrocardiogram evidence, and detection of elevated cardiac troponin in laboratory tests. A questionnaire with objective questions on risk and lifestyle factors such as smoking, sedentary lifestyle, use of diabetes medications, dyslipidemia, hypertension, kidney disease, and family history of coronary artery disease was administered to patients with infarction. Within the first 24h of admission to the service, all patients had their GRACE, TIMI RISK, and KILLIP Scores calculated. During hospitalization, all study subjects underwent echocardiography and cardiac catheterization, the latter procedure being used to calculate the SYNTAX score.

### Ethical considerations

The study Laboratory Model for Stratification of Risk of Intra-hospital Death Associating Hematological Score, Immunological Markers and Metalloproteinases in Patients with Acute Myocardial Infarction. This study was approved by the Ethics in Research Committee in the HUOC/PROCAPE Hospital Complex, University of Pernambuco, number CAAE: 07602819.6.0000.5192 and Report Nº 3.229.657, approval date March 28, 2019. This research followed its ethical guidelines in line with the Declaration of Helsinki. Written informed consent was obtained from all participants.

For the secondary analysis a separate ethical approval was obtained from the Ethics in Research Committee in the HUOC/PROCAPE Hospital Complex, University of Pernambuco number CAAE: 65739322.8.0000.5192 and Report Nº 5.832.237, approval date December 21, 2022. In alignment with Resolution No. 466/2012 of the National Health Council of the Ministry of Health, which provides guidelines and regulatory standards for research involving human beings, patient consent was waived for this research, which uses secondary data. Patients had all identifying information removed, ensuring that their data was anonymized.

### Procedures

The study biomarkers (neutrophils, lymphocytes, red blood cells, and mean platelet volume) were obtained by venipuncture on the first and second days of hospitalization using tubes with ethylene diamine tetraacetic acid (EDTA) anticoagulant and were measured using the Sysmex XE-2100 automated analyzer (Sysmex Europe GmbH, Norderstedt, Germany) [17,18]. The following criteria were adopted: MPV cutoff level ≥ 10.4 femtoliters, NRBC positive was considered as any value other than zero and the NLR was calculated by dividing the neutrophil count by the lymphocyte count, with a cutoff point ≥ 3.7, with the scoring system having a cutoff point of 26 points, allowing patients to be categorized into two groups: low and high risk of death, representing < 26 points and ≥ 26 points respectively [16]. Troponin levels were measured using a Roche analyzer (Elecsys® Troponin T STAT Immunoassay, Roche Diagnostics, Germany) [19]. Cardiac catheterization was performed by a hemodynamicist (interventional cardiologist) and after the examination, the patients had their Syntax Score calculated. Based on the coronary angiograms obtained and using the web calculator, the Syntax Score was obtained by answering questions about the characteristics of coronary artery disease in each patient, with the three initial questions regarding the location of the lesion, the number of lesions and vascular segments affected and nine remaining questions, totaling twelve questions, about the complexity of each lesion, including information about the position, length, tortuosity of each lesion, whether it is located in bifurcations or trifurcations, in addition to other factors such as the presence of thrombi, calcifications, diffuse disease or small vessel caliber [5]. In our study, we classified the anatomical complexity, i.e., the angiographic severity of coronary lesions (Syntax Score) into two ranges: low risk (less than 32 points) and high risk (equal or greater than 32 points).

### Statistical analysis

**Univariate analysis:** involved calculating frequency distributions and central tendencies for each variable, followed by an analysis of variable associations. Data (continuous variables) that were normally distributed were expressed as mean ± standard deviation or median and quartiles for those that were not normally distributed. Absolute (n) and percentage (%) values were used to express categorical variables. **Multivariate analysis:** the two-tailed Pearson chi-square test (χ2) with Yates correlation or Fisher’s exact test were used to compare categorical variables. The Kolmogov-Smirnov test was used to verify the normality of the distribution, then we applied the Student’s t-test or the non-parametric Mann-Whitney test for variables with normal or non-normal distribution, respectively. The Type I error (α) was set at 5%, with results considered statistically significant when p < 0.05 and a 95% confidence level (statistical power). SPSS Statistics, version 10.0, was the software tool used to conduct the statistical analyses.

Sensitivity represented the proportion of patients categorized as high risk by the hematologic score who were classified with a SYNTAX score ≥ 32, which reflects complex coronary lesions. Specificity represents the percentage of patients categorized as low risk by hematologic score who were classified with a SYNTAX score ≤ 32, which reflects patients with less complex coronary artery disease. The Positive Predictive Value (PPV) was the probability of patients presumably at high risk in the hematologic score having a Syntax Score ≥ 32, while the Negative Predictive Value (NPV) is the probability of patients presumably at low risk having a Syntax Score ≤ 32. Accuracy was defined as the proportion of correct predictions (sum of true positives and true negatives) and expresses the degree of certainty in making decisions on patient classification (low or high risk) based on the hematologic score. Accuracy ranges from 0% to 100%, and the higher its value, the greater the possibility of differentiating patients with complex coronary lesions using the proposed score. The respective 95% confidence intervals for these proportions were also calculated. The score performance was analyzed using the receiver operating characteristic (ROC) curve. The area under the curve was used to assist in the differential capacity of the score in question.

## Results

### Patient characteristics

Between June and October 2019, a total of 572 patients were initially screened at our hospital, of whom 93 were excluded. In the end, 479 individuals participated in the study. Table 1 shows the demographic characteristics, risk factors, and clinical history of the participants. The study population was composed of 63% male individuals with a mean age of 62.3 ± 11.1 years, with 78.5% diagnosed with ST-segment elevation myocardial infarction. Among the risk factors, most patients had arterial hypertension (73.1%) and a sedentary lifestyle (66.4%). A total of 457 patients underwent coronary angioplasties (95%) and 22 cardiac surgeries (5%).

**Table 1:** Baseline characteristics of study patients (N = 479).

Characteristics	Statistics
Demographics	
Age (mean ± SD)	62.3 ± 11.1
Sex	
Female	177 (37.0%)
Male	302 (63.0%)
Clinicals history	
Hypertension	350 (73.1%)
Diabetes mellitus	165 (34.4%)
Kidney disease	22 (4.6%)
Dyslipidemia	143 (29.9%)
Smoking history	202 (42.2%)
Family history of heart disease	175 (36.5%)
Depression	41 (8.6%)
Sedentary lifestyle	318 (66.4%)
Acute myocardial infarction	
STEMI	376 (78.5%)
Non-STEMI	103 (21.5%)
Syntax Score	
Low risk	446 (93.1%)
High risk	33 (6.9%)
Death	
Yes	34 (7.1%)
No	445 (92.9%)

Abbreviations: STEMI: with ST elevation myocardial infarction; non-STEMI: withnon-ST elevation myocardial Infarction; NLR: neutrophil to lymphocyte ratio and MPV: mean platelet volume.

Table 2 shows characteristics related to the hematological score and Syntax Score. 166 patients (35%) had a high hematological score and a high Syntax Score for 33 individuals (7%). NRBC was absent in 470 patients (99%). The sensitivity and specificity of the hematologic score were 39.4% and 65.7%, respectively, with a negative predictive value of 93.6%. When the components of the hematologic score were analyzed separately, the MPV showed a sensitivity of 60.6%, a specificity of 46.0% and a negative predictive value of 94.0%; the NLR showed a sensitivity of 63.6% and specificity of 35.7% with a negative predictive value of 93.0%.

**Table 2:** Estimation of hematological parameters MPV and NLR in the definition of angiographic lesions represented by the Syntax score.

Hematological parameters	High risk (n = 33)	Low risk (n = 446)
Hematologic Score (cut-off point of 26)		
High risk (≥ 26)	13	153
Low risk (< 26)	20	293
Sensitivity:	39.4% (35.0 - 43.8)	
Specificity:	65.7% (61.4 - 70.0)	
Positive predictive value:	7.8% (5.4 - 10.2)	
Negative predictive value:	93.6% (91.4 - 95.8)	
Area under the curve (ROC):	50.8% (40.3 - 61.3)	
VPM		
High risk (> 10,4)	20	241
Low risk (≤10,4)	13	205
Sensitivity:	60.6% (56.2 - 65.0)	
Specificity:	46.0% (41.5 - 50.4)	
Positive predictive value:	7.7% (5.3 - 10.0)	
Negative predictive value:	94.0% (91.9 - 96.2)	
Area under the curve (ROC):	56.3% (46.7 - 65.9)	
RNL		
High risk (≥ 3,7)	21	287
Low risk (<3,7)	12	159
Sensitivity:	63.6% (59.3 - 67.9)	
Specificity:	35.7% (31.4 - 39.9)	
Positive predictive value:	6.8% (4.6 - 9.1)	
Negative predictive value:	93.0% (90.7 - 95.3)	
Area under the curve (ROC):	51.3% (39.2 - 63.5)	

NLR: neutrophil to lymphocyte ratio; MPV: mean platelet volume; ROC: Receiver operating characteristic.

**Fig 1:**
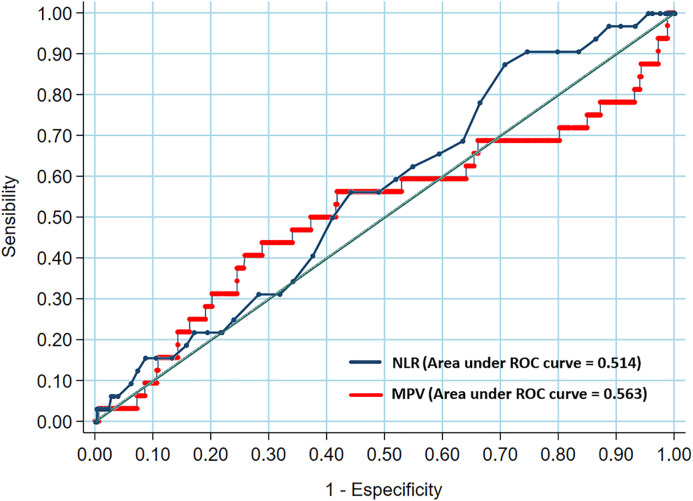
Receiver operating characteristic curve of neutrophil to lymphocyte ratio – NLR/Syntax Score and receiver operating characteristic curve of mean platelet volume – MPV/Syntax Score.

**Fig 2:**
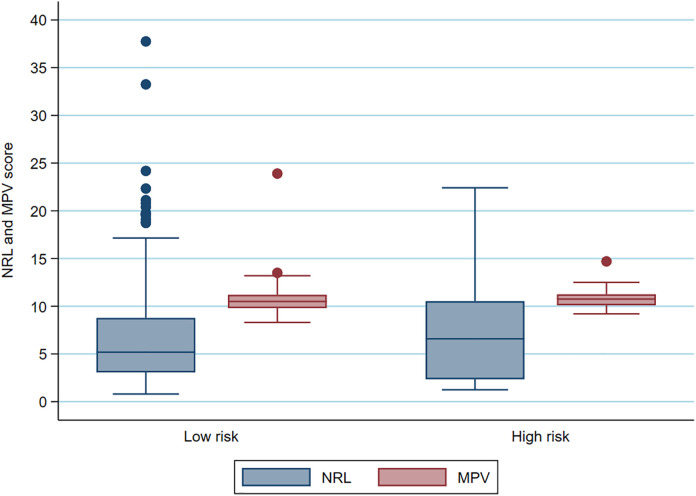
Boxplot to show the association between the NLR/Syntax Score and the association between the MPV/Syntax Score.

In agreement with the literature, the NLR and MPV parameters were associated with the Syntax Score and, in this study, we demonstrated the sensitivity, specificity, positive and negative predictive values (Table 3), C-statistics between these variables (Fig 1) and Boxplot to show the association between the NLR/Syntax Score and the association between the MPV/Syntax Score (Fig 2).

**Table 3:** Combined estimation of hematologic parameters MPV and NLR in the definition of angiographic lesions represented by the Syntax score.

Hematological parameters	High risk (n = 33)	Low risk (n = 446)
NRL or VPM		
High risk (at least one)	27	375
Low risk (both)	6	71
Sensitivity:	81.8% (78.4 - 85.3)	
Specificity:	15.9% (12.6 - 19.2)	
Positive predictive value:	6.7% (4.5 - 9.0)	
Negative predictive value:	92.2% (89.8 - 94.6)	
NRL and VPM		
High risk (both)	14	293
Low risk (at least one)	19	153
Sensitivity:	42.4% (38.0 - 46.8)	
Specificity:	65.7% (61.4 - 70.0)	
Positive predictive value:	8.4% (5.9 - 10.9)	
Negative predictive value:	93.9% (91.8 - 96.1)	

NLR: neutrophil to lymphocyte ratio; MPV: mean platelet volume.

## Discussion

The hematological score with the parameters NRBC, NLR and MPV was proposed with the aim of predicting mortality in patients hospitalized with acute myocardial infarction. Therefore the rationale of the hematological score is to dynamically predict the prognostic predictability of low or high mortality, in a timely manner [15]. However, experimentally, extrapolating the idea of the hematological score scoring scale of 49 points, cut-off of 26 points, in the predictability of low or high complexity of coronary angiographic lesions (Syntax Score), a sensitivity of 39.4%, a specificity of 65.7%, a negative predictive value of 94.6% and an area under the curve of 50.8% were demonstrated.

In this study, the main objective was to evaluate whether there is an association between these hematological score variables and the coronary angiographic lesions represented by the Syntax Score. However, in this sample, NRBC had a very low representation due to the low clinical presentation of hypoxemia and septicemia, the main factors in the release of these immature erythrocyte cells into the peripheral circulation. However, the NLR and MPV variables, as described in the literature, are widely associated with coronary angiographic lesions (Syntax Score).

There are several published studies demonstrating the association of NLR with the complexity of coronary angiographic lesions represented by the Syntax Score. Shunbao Li et al described that the level of NLR in patients with high Syntax Score, which is significantly higher than in patients with the low Syntax Score [20]. Mehdi Maleki et al demonstrated that higher NLR was significantly associated with a higher Syntax Score (beta = 0.162, *P* = 0.021) [21]. Serkan Kurtul et al demonstrated that NLR was significantly lower in patients with a low Syntax Score compared to those with an intermediate or high Syntax Score (3.7 ± 4 to 4.6 ± 2 and 7.9 ± 4, P < 0.001). Linear regression analysis revealed that NLR (coefficient β = 0.380, 95%CI: 1.165–1.917, P < 0.001) was significantly associated with the Syntax Score in patients with NSTEMI [22]. Marco Zuin et al demonstrated that the NLR significantly correlates with Syntax Score with 1-year cardiovascular mortality in patients with ST-segment elevation myocardial infarction (STEMI) or non-ST-segment elevation myocardial infarction (NSTEMI) treated with percutaneous coronary intervention (PCI) within 24-h (OR 2.85, 95% CI 1.54–5.26, p = 0.001 and OR 2.57, 95% CI 1.62–4.07, p < 0.0001 for STEMI and NSTEMI respectively) [23].

Larger and more metabolically active platelets are reflected by MPV, which makes this hematological parameter relevant as a biomarker of atherosclerotic plaque instability and, consequently, of the severity of coronary lesions, represented by the elevated SYNTAX Score. Many studies have described this association between VPM and the Syntax Score. Berkay Ekici et al are quite categorical in clarifying a positive correlation between MPV and Syntax Score (p < 0.001, r = 0.504) [24]. G. Abalı et al described the relationship between MVP and the severity of coronary atherosclerosis in patients with diabetes mellitus and found that MPV has a positive correlation with Syntax Score [25]. Durmus Yıldıray Sahin et al described the association with the extent and complexity of coronary artery disease in diabetic patients with ST elevation myocardial infarction (STEMI) was stronger than for nondiabetic STEMI patients (r ¼.473, P < .001 vs r ¼.129, P ¼.001) [26]. Ioannis Vogiatzis et al demonstrated that Mean Platelet Volume (MPV) is the primary indicator of platelet activation was significantly correlated to Syntax Score (r = 0.658, p < 0.001) and found to be an independent predictor factor of major adverse cardiovascular events (MACE) with HR = 6.8 (95% Confidence Interval (1.46–33.36) [27].

This study evaluated the sensitivity and specificity of NLR and MPV in relation to the Syntax score to predict the angiographic severity of coronary lesions in patients with acute myocardial infarction. NLR and MPV are independent predictors of the severity of coronary angiographic lesions, represented by the Syntax score. Therefore, in this study, the significant presence of NLR or MPV, with values ≥ 10.4 and ≥ 3.7 respectively, represented a good correlation with a high Syntax score (sensitivity 81.8%, specificity 15.9%, negative predictive value 92.2%). However, when NLR and MPV were combined to define a high Syntax score, thus requiring greater diagnostic rigor, the sensitivity dropped to 42.4%, but with an obvious improvement in specificity of 65.7% and negative predictive value of 93.9%. However, most of the study population (93.1%) had less complex coronary artery disease (CAD), and these hematologic variables demonstrated a significant correlation with the Syntax score. Therefore, as a screening method in the emergency room, one of the two elevated variables would already characterize a high Syntax score (sensitivity of 81%), which would be another prognostic tool. However, further studies are needed for effective use in clinical practice.

These hematological parameters (NRBC, NLR, MPV) are a good tool for in-hospital surveillance of all-cause mortality in patients hospitalized with acute myocardial infarction. There is evidence in the literature of the relationship between neutrophil to lymphocyte ratio and mean platelet volume with coronary angiographic lesions (Syntax Score), however such a relationship has not yet been demonstrated with the nucleated red blood cells (NRBC). Coronary angiographic lesions have eminently inflammatory and hematological components, as there is an association between the severity of coronary atherosclerotic plaques (Syntax score) and hematological variables.

Some limitations should be considered in our study, such as those inherent in a single-center study, in addition to the limited sample size, with a small percentage of high syntax scores among the population studied.

## Conclusion

An increase in one of the NLR or MPV parameters, components of the hematologic score (NRBC, NRL, MPV), has been shown to be closely associated with complex coronary angiographic lesions. Furthermore, the hematologic score has been shown to be useful in identifying a population at low risk of coronary artery disease regarding its extent and severity, thus ruling out severe cases. However, further studies are needed to demonstrate the relationship between these hematologic variables and coronary angiographic lesions; because they are easy to measure and inexpensive, they can be readily incorporated into clinical practice.

## References

[1] Matter MA, Paneni F, Libby P, Frantz S, Stähli BE, Templin C, et al. Inflammation in acute myocardial infarction: the good, the bad and the ugly. Eur Heart J. 2024;45(2):89–103. doi: 10.1093/eurheartj/ehad486 37587550 PMC10771378

[2] Dai X-T, Kong T-Z, Zhang X-J, Luan B, Wang Y, Hou A-J. Relationship between increased systemic immune-inflammation index and coronary slow flow phenomenon. BMC Cardiovasc Disord. 2022;22(1):362. doi: 10.1186/s12872-022-02798-0 35941535 PMC9358856

[3] Vukicevic P, Klisic A, Neskovic V, Babic L, Mikic A, Bogavac-Stanojevic N, et al. New markers of platelet activation and reactivity and oxidative stress parameters in patients undergoing coronary artery bypass grafting. Oxid Med Cell Longev. 2021;2021:8915253. doi: 10.1155/2021/8915253 34257821 PMC8257340

[4] Sikora-Frac M, Zaborska B, Maciejewski P, Budaj A, Bednarz B. Improvement of left ventricular function after percutaneous coronary intervention in patients with stable coronary artery disease and preserved ejection fraction: Impact of diabetes mellitus. Cardiol J. 2021;28(6):923–31. doi: 10.5603/CJ.a2019.0066 31257568 PMC8747832

[5] Serruys PW, Morice M-C, Kappetein AP, Colombo A, Holmes DR, Mack MJ, et al. Percutaneous coronary intervention versus coronary-artery bypass grafting for severe coronary artery disease. N Engl J Med. 2009;360(10):961–72. doi: 10.1056/NEJMoa0804626 19228612

[6] Hashmi SA, Khowaja R, Ali M, Mangi AR, Khowaja A, Riaz G, et al. Prognostic significance of nucleated RBCs in predicting mortality among ST-elevation myocardial infarction patients admitted to the ICU. Cureus. 2023;15(9):e45445. doi: 10.7759/cureus.45445 37859905 PMC10583491

[7] Chen Z, He Y, Su Y, Sun Y, Zhang Y, Chen H. Association of inflammatory and platelet volume markers with clinical outcome in patients with anterior circulation ischaemic stroke after endovascular thrombectomy. Neurol Res. 2021;43(6):503–10. doi: 10.1080/01616412.2020.1870359 33402058

[8] Agarwal R, Aurora RG, Siswanto BB, Muliawan HS. The prognostic value of neutrophil-to-lymphocyte ratio across all stages of coronary artery disease. Coron Artery Dis. 2022;33(2):137–43. doi: 10.1097/MCA.0000000000001040 33826535

[9] Morici N, Molinari V, Cantoni S, Rubboli A, Antolini L, Sacco A, et al. Long-term risk of major adverse cardiovascular events in patients with acute coronary syndrome: prognostic role of complete blood cell count. Angiology. 2020;71(9):831–9. doi: 10.1177/0003319720938619 32638621

[10] Tadesse S, Gudina EK, Yilma D, Asefa ET, Yemane T, Mossie A. Haematological indices in acute coronary syndrome patients in Ethiopia: a comparative cross-sectional study. J Blood Med. 2024;15:275–84. doi: 10.2147/JBM.S457371 38912419 PMC11193461

[11] Zhao Y, Zhang S, Yi Y, Qu T, Gao S, Lin Y, et al. Neutrophil-to-lymphocyte ratio as a predictor for cardiovascular diseases: a cohort study in Tianjin, China. J Hum Hypertens. 2023;37(7):576–83. doi: 10.1038/s41371-022-00724-7 35859165

[12] Tavares F, Moraes PIM, Souza JM, Barbosa AH, Santos EM, Marcondes JA, et al. Prognostic role of neutrophil-to-lymphocyte ratio in patients with ST-elevation myocardial infarction undergoing to pharmaco-invasive strategy. Cardiovasc Revasc Med. 2022;34:99–103. doi: 10.1016/j.carrev.2021.01.027 33736961

[13] Mangalesh S, Dudani S, Mahesh NK. Development of a novel inflammatory index to predict coronary artery disease severity in patients with acute coronary syndrome. Angiology. 2024;75(3):231–9. doi: 10.1177/00033197231151564 36629740

[14] Yasak IH, Tascanov MB, Gönel A, Seyhanli ES. The relationship between the severity of coronary artery disease and erythrocyte morphology parameters measured by new-generation hematology analyzer. Comb Chem High Throughput Screen. 2022;25(8):1278–83. doi: 10.2174/1386207324666210528113024 34053423

[15] Monteiro Júnior JG de M, Torres D de OC, da Silva MCFC, Martins CM de H, da Silva IK, do Nascimento MEM, et al. Prognostic value of hematological parameters in patients with acute myocardial infarction: intrahospital outcomes. PLoS One. 2018;13(4):e0194897. doi: 10.1371/journal.pone.0194897 29668734 PMC5905886

[16] Monteiro Júnior JG de M, Torres D de OC, Silva MCFC da, Príncipe TRN, Vasconcelos RB de, Brito MEC de, et al. Performance of a hematological scoring system in predicting all-cause mortality in patients with acute myocardial infarction. Int J Cardiovasc Sci. 2020;33(4):380–8. doi: 10.36660/ijcs.20190094

[17] Nakul-Aquaronne D, Sudaka-Sammarcelli I, Ferrero-Vacher C, Starck B, Bayle J. Evaluation of the Sysmex Xe-2100 hematology analyzer in hospital use. J Clin Lab Anal. 2003;17(4):113–23. doi: 10.1002/jcla.10083 12784259 PMC6807756

[18] Hwang DH, Dorfman DM, Hwang DG, Senna P, Pozdnyakova O. Automated nucleated RBC measurement using the Sysmex XE-5000 hematology analyzer: frequency and clinical significance of the nucleated RBCs. Am J Clin Pathol. 2016;145(3):379–84. 26834126 10.1093/ajcp/aqv084

[19] Del Carlo CH, Pereira-Barretto AC, Cassaro-Strunz CM, Latorre M do RD de O, Oliveira Junior MT de, Ramires JAF. Troponina cardíaca T para estratificação de risco na insuficiência cardíaca crônica descompensada. Arq Bras Cardiol. 2009;92(5):404–12. doi: 10.1590/S0066-782X2009000500012 19629294

[20] Li S, Chen H, Zhou L, Cui H, Liang S, Li H. Neutrophil-to-lymphocyte ratio predicts coronary artery lesion severity and long-term cardiovascular mortality in patients with unstable angina pectoris. Acta Cardiol. 2022;77(8):708–15. doi: 10.1080/00015385.2021.1963564 35969267

[21] Maleki M, Tajlil A, Separham A, Sohrabi B, Pourafkari L, Roshanravan N, et al. Association of neutrophil to lymphocyte ratio (NLR) with angiographic SYNTAX score in patients with non-ST-Segment elevation acute coronary syndrome (NSTE-ACS). J Cardiovasc Thorac Res. 2021;13(3):216–21. doi: 10.34172/jcvtr.2021.40 34630969 PMC8493237

[22] Kurtul S, Sarli B, Baktir AO, Demirbas M, Saglam H, Doğan Y, et al. Neutrophil to lymphocyte ratio predicts SYNTAX score in patients with non-ST segment elevation myocardial infarction. Int Heart J. 2015;56(1):18–21. doi: 10.1536/ihj.14-175 25742940

[23] Zuin M, Rigatelli G, Picariello C, dell’Avvocata F, Marcantoni L, Pastore G, et al. Correlation and prognostic role of neutrophil to lymphocyte ratio and SYNTAX score in patients with acute myocardial infarction treated with percutaneous coronary intervention: a six-year experience. Cardiovasc Revasc Med. 2017;18(8):565–71. doi: 10.1016/j.carrev.2017.05.007 28529092

[24] Ekici B, Erkan AF, Alhan A, Sayın I, Aylı M, Töre HF. Is mean platelet volume associated with the angiographic severity of coronary artery disease? Kardiol Pol. 2013;71(8):832–8. doi: 10.5603/KP.2013.0195 24049023

[25] Abalı G, Akpınar O, Söylemez N. Correlation of the coronary severity scores and mean platelet volume in diabetes mellitus. Adv Ther. 2014;31(1):140–8. doi: 10.1007/s12325-013-0081-9 24318519

[26] Sahin DY, Gür M, Elbasan Z, Özdoğru I, Uysal OK, Kivrak A, et al. Mean platelet volume and extent and complexity of coronary artery disease in diabetic and nondiabetic patients with ST elevation myocardial infarction. Angiology. 2013;64(7):505–11. doi: 10.1177/0003319712460423 23028178

[27] Vogiatzis I, Samaras A, Grigoriadis S, Sdogkos E, Koutsampasopoulos K, Bostanitis I. The mean platelet volume in the prognosis of coronary artery disease severity and risk stratification of acute coronary syndromes. Med Arch. 2019;73(2):76–80. doi: 10.5455/medarh.2019.73.76-80 31391691 PMC6643353

